# Broad Spectrum Anti-Influenza Agents by Inhibiting Self-Association of Matrix Protein 1

**DOI:** 10.1038/srep32340

**Published:** 2016-08-30

**Authors:** Philip D. Mosier, Meng-Jung Chiang, Zhengshi Lin, Yamei Gao, Bashayer Althufairi, Qibing Zhou, Faik Musayev, Martin K. Safo, Hang Xie, Umesh R. Desai

**Affiliations:** 1 Department of Medicinal Chemistry and Institute for Structural Biology, Drug Discovery and Development, School of Pharmacy, Virginia Commonwealth University, Richmond, Virginia, United States of America; 2 Division of Viral Products, Office of Vaccines Research and Review, Center for Biologics Evaluation and Research, United States Food and Drug Administration, Silver Spring, Maryland, United States of America; 3 Department of Nanomedicine & Biopharmaceuticals, National Engineering Research Center for Nanomedicine, Huazhong University of Science and Technology, Wuhan, Hubei, China

## Abstract

The matrix protein 1 (M1) of influenza A virus (IAV) exists as a three-dimensional oligomeric structure in mature virions with high sequence conservation across different IAV subtypes, which makes it a potential broad spectrum antiviral target. We hypothesized that impairing self-association of M1 through a small molecule ‘wedge’, which avidly binds to an M1-M1 interface, would result in a completely new class of anti-influenza agents. To establish this proof-of-principle, we performed virtual screening on a library of >70,000 commercially available small molecules that resulted in several plausible ‘wedges’. Biophysical studies showed that the best molecule bound the M1 protein potently and weakened M1-M1 self-association. Most importantly, the agent reduced the thickness of the M1 layer in mature virions and inhibited *in ovo* propagation of multiple IAV strains including H1N1, pandemic H1N1, H3N2 and H5N1, which supports the “wedge” hypothesis. These results demonstrate that M1 is a promising druggable target for the discovery of a completely new line of broad spectrum anti-IAV agents.

It is estimated that more than 35 million cases of influenza-like illness have occurred in the US alone in a single season (2013–2014)^1^ despite the availability of multiple seasonal influenza vaccines. This is due to the rapid mutation of viral surface glycoproteins hemagglutinin (HA) and neuraminidase (NA) resulting in frequent antigenicity changes^2^, which makes annual influenza vaccine strain selection difficult to match with circulating viruses^3^. In recent years, tremendous efforts have been focused on developing universal vaccines that target a variety of conserved epitopes in HA to induce broadly neutralizing antibodies^4^,^5^,^6^. In addition, alternative approaches that target the virus’s internal proteins such as matrix protein 1 (M1) and nucleoprotein (NP) have also been pursued for promoting cross-reactive T cell immunity^7^,^8^. However, developing universal vaccines is extremely challenging due to the high plasticity of influenza A virus (IAV), of which 18 HA subtypes and 11 NA subtypes are known today. With increased activities of highly pathogenic avian influenza virus detected globally^9^,^10^,^11^, there is an urgent demand for effective counter-measures including broad spectrum antivirals for pandemic preparedness.

Current anti-IAV drugs target either the M2 ion channel (adamantine and rimantidine) or NA (oseltamivir, zanamivir and peramivir) of the viral envelope. However, the rapid evolution of NA and to a lesser extent M2^12^,^13^, as well as the widespread resistance to M2 inhibitors^14^,^15^,^16^,^17^,^18^, underscore the urgency for better antiviral agents. Efforts to discover such agents have employed drug design strategies based on either mechanistic (e.g., Kim *et al*.^19^) or structural (e.g., Massari *et al*.^20^) paradigms. Such strategies are being earnestly pursued against HA, NA, M2, nonstructural protein, RNA polymerases, and NP^21^,^22^, which represent all, but one, proteins of the IAV. The one protein of IAV against which no inhibitor has been developed to date is the M1 protein.

The M1 protein forms a three-dimensional layer underneath the lipid bilayer of the virion serving as a bridge to connect the membrane proteins including HA and NA with the internal viral ribonucleoprotein (vRNP) core^23^. The M1 protein is critical for structural and functional integrity of the mature virion^24^ and is involved in multiple replication steps including virion uncoating, nuclear export of the vRNP complex, and assembly and budding of newly formed viral particles^23^. These pleotropic, but highly coordinated, roles of M1 possibly arise from its propensity to assume multiple conformational states dependent on the local conditions, such as pH and lipid binding^25^,^26^,^27^,^28^,^29^,^30^,^31^. In fact, self-association or oligomerization (to maintain the integrity of the viral capsid at neutral pH) and de-oligomerization (to release the vRNPs in the late endosome at low pH) of the M1 layer are essential for viral replication^32^.

At a molecular level, the M1 polypeptide monomer displays a three-dimensional surface that can be visualized as a ‘brick’ (Fig. 1). Self-association of M1 results in the formation of the M1 layer in mature virions, which is an elongated, non-covalent polymer underneath the envelope membrane that is critical for viral integrity. We reasoned that reducing the stability of the M1 layer by disrupting the self-association of M1 ‘bricks’ using an appropriate small molecule (or ‘wedge’) could be a powerful strategy to discover a completely new line of antiviral agents. ‘Wedges’ that bind to at least one of the M1-M1 interfaces could interfere with M1 layer formation resulting in deformed viral particles with impaired replication potential. This novel approach of targeting M1 for drug discovery is a high-risk venture because it involves disrupting the interaction of a large interface with a comparatively very small agent^33^,^34^. However, with regard to M1 it may have some advantages. An appropriate small molecule need not completely prevent M1-M1 self-association, but alter it just enough to reduce the stability of the M1 layer. A second advantage, especially with regard to druggability of the target protein, is that self-association to form the M1 layer presents multiple sites of binding for a complementary ‘wedge’, which enhances the probability of destabilization of the M1 layer by a small molecule. Finally, a most important advantage is that the M1 protein is relatively more conserved than other IAV proteins^15^,^17^,^35^ (see below), which can be expected to minimize the impact of selection pressures (either environmental or drug-induced).

## Results and Discussion

### Genetic Basis for Targeting M1

The M1 protein is one of the most highly conserved proteins in the IAV^35^. It has been shown to evolve at a much slower rate than even its cognate M segment product, the M2 proton channel^17^. Analysis of a large set of M1 and M2 sequences (*vide supra*) corroborates these findings. The M1 sequences show 29.8% identity and 57.5% similarity, whereas M2 sequences show only 2.1% identity and 20.6% similarity (Supplementary Fig. 1, 2, 3 and 4). More strikingly, mutations are extremely rare at many positions in M1. Very few sequences out of the hundreds possess a divergent amino acid residue, which results in a sequence conservation rate of nearly 100%. Further, the positions of lowest sequence identity in M1 are dominated by a few homologous amino acid residues (e.g., 15:V/I; 95:R/K; 101:R/K; 121:A/T; 142:V/A/G; 166:V/A; and 227:A/T). In contrast, significantly higher stereo-electronic variability is observed for positions of lowest sequence identity in M2 (e.g., 18:R/K/N; 28:V/I/D/T).

**Figure 1:**
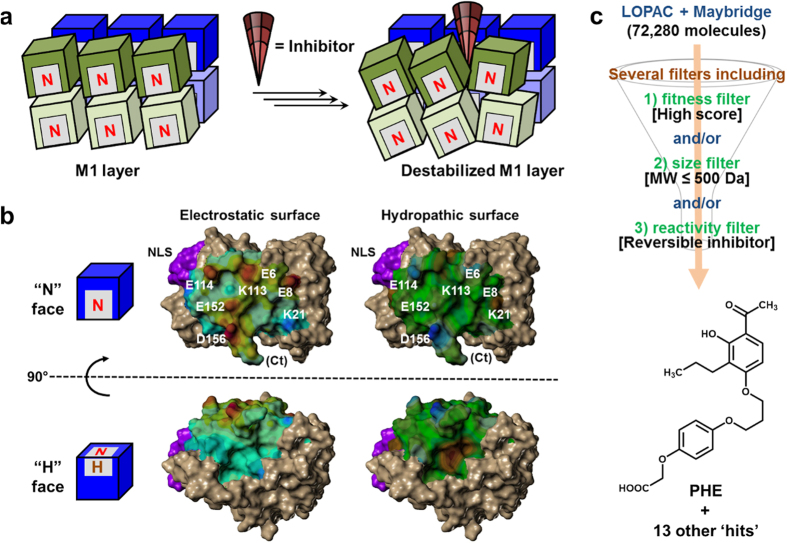
A putative mechanism of M1 layer disruption by small molecules. (**a**) Disruption of the M1 layer by a small-molecule ‘wedge’. Blue and green blocks represent adjacent M1 dimers, gray patches represent the binding site that extends across two faces of M1, and the red cone represents the disrupting small-molecule ‘wedge’. (**b**) The crystal structure of the M1 N-terminal domain at neutral pH (PDB ID = 1EA3). An M1 binding site consisting of the negatively charged (“N”) face and its adjacent hydrophobic pocket (“H”) face was studied for discovering wedges. Shown are electrostatic (blue = positive; red = negative) and hydropathic (blue = hydrophilic; brown = hydrophobic) surfaces. The nuclear localization signal (NLS; ^101^RKLKR^105^) motif is shown in magenta. (**c**) The virtual screening algorithm used to identify the lead compound PHE.

Although M1’s high degree of conservation could be attributed to low evolutionary selection pressure compared to other IAV proteins (most notably HA and NA, which are known to mutate at a very high rate), it may also mean that sequence variation may compromise its ability to form a confluent protein coat, which is critical for other functions, such as stabilizing the viral envelope during membrane fusion^23^ and the nuclear export of viral ribonucleoproteins^36^. Thus, changes in the M1 sequence may be detrimental to the virus. This implies that disruption of M1 self-association by either kinetic or thermodynamic means will likely produce a deformed M1 layer, which might reduce viral replication potential. We posited that such disruption could be induced by small molecules that bind to one or more M1-M1 interfaces (Fig. 1).

### Virtual Screening Identifies a Number of Candidates That Could Target M1

To discover destabilizers of the M1 layer formation, we studied the nature of forces that support M1-M1 interaction using published crystal structures under different conditions^25^,^27^,^29^,^30^,^31^. An M1 ‘brick’ interacts with a neighboring M1 through a combination of electrostatic and hydrophobic forces^26^,^29^. One face of the brick, which includes the basic nuclear localization signal (NLS; ^101^RKLKR^105^) in one corner (designated here as the ‘P’ face), is positively charged, while the opposing face contains a collection of acidic residues (the ‘N’ face), thus favoring complementarity (Fig. 1b). Interestingly, a face containing a hydrophobic pocket (the ‘H’ face) is found adjacent to the ‘N’ and ‘P’ faces, which may serve as a “hot spot” for a small molecule to bind. We hypothesized that a ‘wedge’ could be identified by targeting such a hot spot in the neighborhood of the ‘N’ and ‘H’ faces (Fig. 1a). To identify such ‘wedges’, a virtual screening approach was employed using two drug-like libraries (Maybridge and LOPAC, 72,280 small molecules; Fig. 1c) resulting in the identification of several ‘hits’ (see Supplementary Figs 5 and 6 for all structures).

### *In Vitro* Studies Identify a Promising Anti-IAV Agent

**Figure 2:**
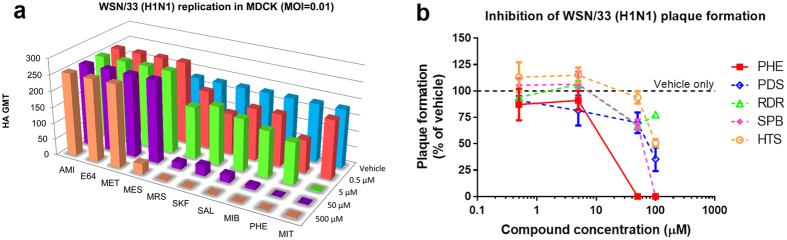
*In vitro* inhibition of virus replication. (**a**) Among the top ten virtual screening hits, six compounds including PHE cause a dose-dependent reduction in HA geometric mean of titer (GMT) of H1N1 A/WSN/33 (WSN/33) replicated in MDCK cells (n = 2–6 replicates). (**b**) Only PHE significantly reduces WSN/33 plaque formation on MDCK monolayer (n = 3 replicates). See Methods for detailed procedure.

For initial screening, Madin-Darby Canine Kidney (MDCK) cells infected with H1N1 A/WSN/33 (WSN/33) were cultured with individual hit compounds at various concentrations. Forty-eight hours later, the supernatants were harvested for hemagglutination (HA) assay. Among the ten hit compounds identified from the first library, six (MRS, MIB, SAL, SKF, PHE and MIT) showed dose-dependent inhibition of *in vitro* replication of WSN/33, whereas the remaining four compounds (AMI, E64, MET and MES) had no obvious antiviral effects (Fig. 2a). Of the six inhibitors identified, MRS, MIB, SAL, SKF and MIT exhibited substantial cytotoxicity (Supplementary Fig. 10a). In contrast, PHE not only showed a high antiviral activity (Fig. 2a) but also possessed minimal cytotoxicity (Supplementary Fig. 10a). Four more compounds (PDS, RDR, SPB and HTS) were identified by screening the second library but each was found to be less effective than PHE at blocking WSN/33 plaque formation (Fig. 2b). Structurally, PHE is a small hydrophobic molecule of molecular weight ~400 that can be chemically synthesized in a few steps. Thus, it represented a promising molecule to further establish the concept of anti-IAV activity through inhibition of M1 self-association.

### Molecular Modeling Suggests PHE Binds to More Than One Site on M1

The initial virtual screening exercise relied on identifying agents that bind at a specific M1-M1 interface site, which was deemed promising from the perspective of discovering potential protein–protein interaction (PPI) inhibitors. The *in vitro* screening success with PHE implies that it may bind at the site predicted by the molecular modeling. It is possible that PHE also binds to alternative sites/interfaces, especially because it is a carboxylic acid derivative that possesses substantial flexibility as well as hydrophobic character, features that are complementary to multiple putative M1 binding sites. To assess this potential, we performed a comprehensive ‘blind’^37^ docking study.

Forty-one overlapping binding sites were defined around basic, acidic, or hydrophobic side chains so as to cover the entire M1 surface. By utilizing a binding site identification strategy that relies on multiple scoring functions, which minimizes bias^38^, we identified four unique sites in addition to the original targeted site that afford favorable PHE binding characteristics (Supplementary Table 1 and Supplementary Figs 7 and 9). These sites were localized either directly on, or adjacent to, the ‘N’ and ‘P’ faces. Interestingly, one of the sites was found to be directly opposite to the initially identified site (Supplementary Fig. 8). Instructively, several studies have been reported on the role of some of residues likely to be present in the region surrounding the putative PHE binding site including K95, K98, R101, K102, K104 and R105^24^,^39^,^40^,^41^. These studies indicate that mutations at these positions significantly affect IAV morphology, replication and/or pathogenicity of the IAV, which support the expectation that PHE binding will disrupt M1-M1 interaction resulting in a deformed M1 layer.

In a traditional drug discovery process, such promiscuity would be viewed as a limitation for a putative drug candidate. However, in the present context it is likely to be an advantage because multiple binding sites could engage more than one PHE molecule, thereby enhancing the probability of M1 layer destabilization (Supplementary Figs 7 and 9). Additionally, multi-site binding may also enhance the apparent affinity of the small molecule. Thus, the comprehensive modeling study indicates that PHE is likely to be a good candidate to critically assess the rationale of targeting M1 for the discovery of potent anti-IAV agents.

### Biophysical Studies Show PHE Binds to M1 and Prevents its Oligomerization

**Figure 3:**
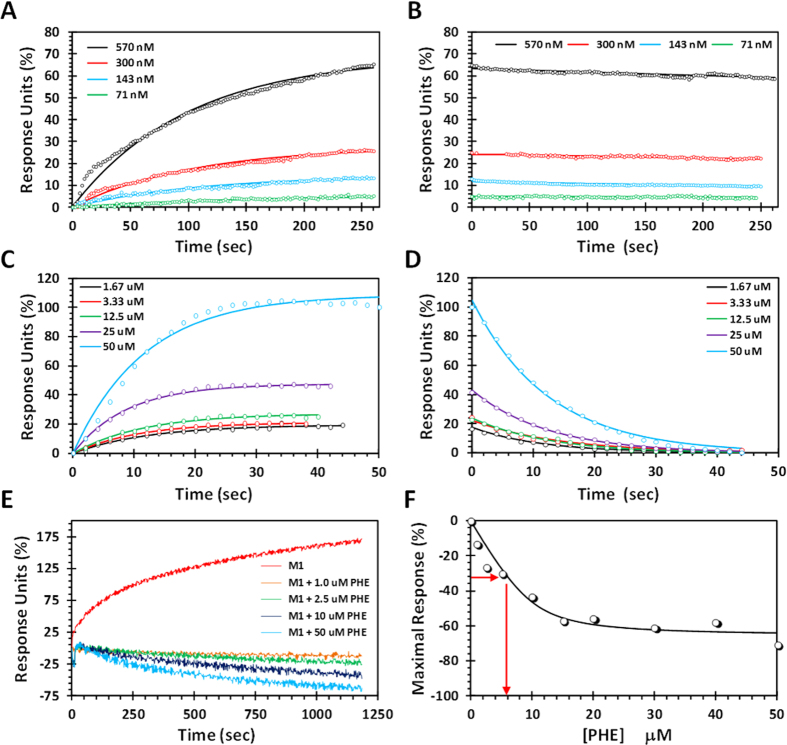
PHE inhibits M1-M1 association. The interaction of M1 with PHE was followed by SPR using biotinylated M1 chips. Panels (**a**,**b**) show the association and dissociation phases of the M1–M1 interaction, respectively, while (**c**,**d**) show the association and dissociation phases of the M1–PHE interaction, respectively. (**e**) Bio-layer interferometry (BLI) response curves for M1 self-association with varying concentrations of PHE. (**f**) A plot of maximal BLI response *versus* PHE concentration projecting an IC_50_ value of ~6 μM.

To test whether PHE binds directly to M1, we utilized surface plasmon resonance (SPR)-based interaction studies (see Methods). M1 was immobilized on NeutrAvidin-gold chips using a biotinylated form of the protein. The association of soluble M1 with chip-bound M1 followed the expected binding profile at pH 7.4 (Fig. 3a). In contrast, the dissociation of M1 from bound M1 was much slower indicating that self-association is an off-rate driven process (Fig. 3b). Considering that M1 in solution may exist in dimeric or oligomeric forms^29^, the SPR kinetics lead to an apparent affinity of 50 ± 30 pM. The M1–PHE interaction was studied in a similar manner and displayed much faster association and dissociation rates (Fig. 3c,d). The ratio of off- to on-rate of interaction led to an affinity of 870 ± 150 nM at pH 7.4 for PHE binding to monomeric M1. This indicates a fairly tight binding interaction for a molecule as small as PHE.

We then turned to bio-layer interferometry using Octet Qke equipped with streptavidin biosensor tips to assess whether PHE impairs M1 self-association (see Methods). This technique is particularly suited to assess oligomerization process as the signal is highly sensitive to changes in molecular weight of the tip-bound target. The increase in molecular weight following M1-M1 self-association shifted the wavelength of interference by more than 175% (Fig. 3e). In contrast, M1 in the presence of 1 to 50 μM PHE displayed reduced signal corresponding to a weakened oligomerization process. The plot of maximal response *versus* PHE concentration displayed a classic hyperbolic relationship, which gave an *IC*_50_ of ~6 μM and a maximal decrease in M1 chain extension of ~60% (Fig. 3f). This indicates that PHE disrupts M1 oligomerization at pH 7.4 with fairly high potency and efficacy.

### PHE Potently Disrupts M1 Layer Formation in Assembled Virions and Inhibits Replication of Multiple IAV Strains *In Ovo*

**Figure 4:**
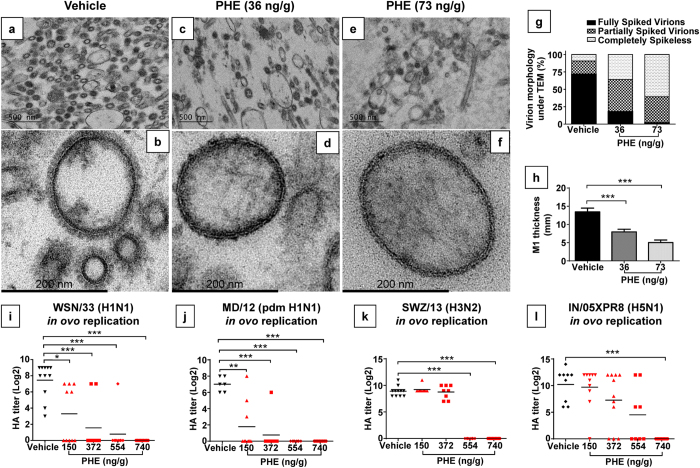
*In ovo* disruption of the M1 layer formation and inhibition of viral replication. TEM images of H1N1 A/WSN/33 (WSN/33) at low magnification in panels (**a**,**c**,**e**) and at high magnification in corresponding panels (**b**,**d**,**f**) show that the morphology of virions purified from vehicle-treated control eggs (**a**,**b**) is different from those purified from PHE (**c**,**d**: 36 ng/g; **e**,**f**: 73 ng/g) co-injected eggs. (**g**) Percent distributions of fully spiked, partially spiked and completely stripped WSN/33 virions purified from eggs with or without PHE treatments. (**h**) PHE reduces the thickness of the M1 layer in WSN/33 viral particles in a dose-dependent manner. PHE also induces a dose-dependent reduction in HA geometric mean of titer (GMT, black lines) of different IAV strains propagated in embryonic eggs, including (**i**) H1N1 A/WSN/33 (WSN/33), (**j**) pandemic H1N1 A/Maryland/13/2012 (pdm H1N1 MD/12), (**k**) H3N2 A/Switzerland/9715293/2013 (SWZ/13), and (**l**) H5N1 vaccine reassortant A/Indonesia/05/2005XPR8 (IN/05XPR8). One-way ANOVA was performed to compare the differences between vehicle only and PHE treatments. HA titers were log-transformed before the analysis. *Indicates *P* < 0.05; **indicates *P* < 0.01; ***indicates *P* < 0.001.

Next we sought to assess PHE’s anti-IAV potential in an *in vivo* model. As a selective agonist of the nuclear transcription factor–peroxisome proliferator-activated receptor β/δ (PPARβ/δ), PHE has both pro-inflammatory and anti-inflammatory effects on host intermediary metabolism and immune regulation^42^, which can theoretically jeopardize our efforts to prove M1 as a druggable target. To overcome PHE-associated complications, we utilized embryonated hen’s egg, which is a preferred matrix for amplifying influenza viruses^43^ because of absence of mature immune defense. We co-injected WSN/33 with PHE into 10-day-old specific-pathogen-free embryonic eggs and then purified WSN/33 amplified in allantoic fluids for transmission electron microscopy (TEM). In the absence of PHE (vehicle only), WSN/33 mature virions were well-formed and of uniform size (Fig. 4a). More than 70% of these virions had a full complement of glycoproteins (spikes) on the surface (Fig. 4b,g). In contrast, virions prepared from PHE co-injected eggs were not only significantly lower in density but also much more variable in size (Fig. 4c,e). PHE co-injection also dramatically altered the morphology of WSN/33 viral particles (Fig. 4d,f vs b). In the presence of PHE, the frequency of fully spiked mature virions fell below 18%, while >82% of viral particles were either partially spiked or completely deprived of spikes (Fig. 4g). Under high magnification, the M1 layer in WSN/33 mature virions with vehicle only appeared as a clearly defined dark band underneath heavily spiked envelope (Fig. 4b). In contrast, the viral particles prepared from PHE co-injected eggs were either loosely coated (Fig. 4d) or completely stripped with a much thinner M1 layer underneath (Fig. 4f). Indeed, the thickness of the M1 layer was substantially reduced by PHE in a concentration-dependent manner (Fig. 4h). In the viral particle, envelope proteins HA and NA *via* their cytoplasmic tails interact with the M1 layer^23^,^44^. PHE-induced destabilization of the M1 layer is likely to disfavor optimal interaction with HA/NA leading to the loss of spikes on viral particles. Thus, consistent with the biophysical data (Fig. 3), these results clearly indicate PHE is able to disrupt M1 layer formation in assembled virions resulting in the loss of strong hold of surface HA and NA.

The consequence of this impairment is that both initiation of infection, which is mediated by HA, and release and spread of newly synthesized viruses, which are mediated by NA^45^, are made dysfunctional leading to inefficient virus replication. Indeed, PHE blocked the *in ovo* replication of WSN/33 in a dose-dependent manner (Fig. 4i). This antiviral activity of PHE was not due to cytotoxicity since all chicken embryos survived at the highest dose of 740 ng/g tested (Supplementary Fig. 10b). More significantly, PHE was also able to block the *in ovo* replication of different IAV strains including pandemic H1N1 A/Maryland/13/2012 (Fig. 4j), H3N2 A/Switzerland/9715293/2013 (Fig. 4k) and A/Fiji/2015 (Supplementary Fig. 11a), and H5N1 vaccine reassortants A/Indonesia/05/2005XPR8 (Fig. 4l) and A/Egypt/N03072/2010XPR8 (Supplementary Fig. 11b), all in a dose-dependent manner regardless of surface glycoproteins. For the viral strains studied, a high dose of ~740 ng/g was found to be sufficient to prevent 90% *in ovo* replication. These results support the principle that targeting M1 with small molecules such as PHE can generate broad spectrum anti-IAV activity.

## Summary

This work demonstrates a novel principle that small hydrophobic molecules with drug-like properties (e.g. PHE MW~400; logD~1.5) can be potent anti-IAV agents (>90% inhibition at 740 ng/g) by impairing the process of M1 oligomerization. This anti-IAV drug design strategy—‘wedge’ disruption of M1 layer formation—belongs to the category of protein–protein interaction (PPI) inhibitors. Although several designed small molecules have been shown to successfully inhibit other PPIs^33^,^34^, e.g., small molecule inhibitors of cIAP/SMAC, bromodomain/histone, MDM2/p53 systems, this is the first report to our knowledge of their successful design with regard to type A influenza virus.

Although belonging to the category of PPIs, PHE belongs to a slightly different type of PPI. Whereas traditional PPIs inhibit formation of complex between one protein molecule with another protein molecule, PHE inhibits formation of the M1 layer, which involves a large number of M1 molecules. Inhibiting self-association, which could be thought of as a non-covalent polymerization process, is difficult. For a molecule as small as PHE (MW <500), the *in vitro* apparent affinity of 1 μM is therefore very promising. This potency decreases significantly in viral replication assays (50 μM) most probably due to factors such as efficacy of host cell penetration, binding to proteins such as ovalbumin, etc. However, such loss in potency when moving to *in vivo* systems is also found for most drugs suggesting that a clinically relevant PHE-like drug may be realized through our new ‘wedge’ design strategy.

Although PHE was demonstrated to be a promising anti-IAV agent in this study, literature reports that PHE influences host intermediary metabolism, inflammation and immune regulation^42^. These effects on the host may limit PHE’s clinical development. Yet, the identification of PHE as the first M1-targeting molecule implies that clinically relevant anti-IAV agents are possible to develop using the ‘wedge’ design strategy. Although PHE most probably targets multiple sites on M1, appropriate analogs of PHE may be possible to design so as to uniquely target an M1 interface/site. In fact, PHE affords a number of opportunities for developing advanced agents through structural modifications of its functional groups such as phenolic OH, carboxylic acid and ethyl substitution. Overall, considering that M1 is highly conserved across IAV strains and the M1 ‘wedge’ design is a unique principle, this work puts forward a completely new paradigm of targeting M1 to derive broad spectrum antivirals with far-reaching clinical benefits in the near future.

## Methods

### Sequence Alignment

Primary amino acid sequences corresponding to the M1 protein and the M2 proton channel were downloaded from the NCBI Influenza Resource Database^46^ (http://www.ncbi.nlm.nih.gov/genomes/FLU/FLU.html). Unless otherwise noted, default settings were used. The ‘Protein’ option was set to either M1 or M2 as necessary. The ‘Full-length only’ option was set to exclude fragments. To ensure that an equal number of M1 and M2 sequences were included in the initial search, only those sequences that were part of a complete influenza genome were considered by checking the ‘Select all’ option in the ‘Required segments’ section. Identical sequences were culled from the list using the ‘Collapse identical sequences’ option. The two M1 sequence data sets were then merged, sorted and duplicate sequences removed. Finally, M1 and M2 sequences with undefined amino acids (‘X’) were removed. The resulting 742 M1 and 1282 M2 sequences represent a large time span (1925–2015) and diverse geographic locations, hosts, and strains (including highly pathogenic avian H5N1, pandemic-associated swine H1N1 and seasonal H3N2). Each set of sequences was then aligned in ClustalX 2.0^47^ using default parameters. Percent identity was calculated in the usual way and percent similarity was calculated using the formula (*I* + *S*)/*T*, where *I* is the number of fully conserved (i.e. identical) positions, *S* is the number of positions that exhibit ‘strong’ conservation (as defined in ClustalX), and *T* is the total number of sequence positions (*T* = 252 for M1 and 97 for M2). The aligned sequences were analyzed using JProfileGrid 2.0^48^.

### Virtual Screening

The crystal structure of the M1 N-terminal N^1–165^ domain of H1N1A/WSN/33 determined at pH 5.5 (PDB = 4PUS; chain A)^29^ and the crystal structure of the M1 N-terminal N^1–164^ domain of H1N1 A/PR/8/34 determined at pH 7 (PDB ID = 1EA3; chain B)^25^ were used to virtually screen compound libraries. For the 4PUS structure, the conformation of the C-terminal residue Q158 side chain was modified by setting its torsion angles (χ_1_ = −177°; χ_2_ = 65°; χ_3_ = 60°) to those of a rotamer from the Penultimate Rotamer Library^49^ that allowed access to the hydrophobic pocket on the “H” face from the electronegative “N” face, similar to the 1EA3 structure (see main text for details). SYBYL-X 2.1 (Certara Inc., St. Louis, MO) was used to add hydrogen atoms to the crystal structures, generate Connolly surfaces and the corresponding electrostatic (Gasteiger–Hückel charges) and lipophilic (computation method = protein; Crippen table = Ghose *et al*.1998) potential maps (Fig. 1b).

Virtual libraries of ready-to-dock structures representing the LOPAC and Maybridge databases were downloaded from ZINC (http://zinc.docking.org)^50^. Virtual screening was accomplished using GOLD Suite 5.1^51^ and the Goldscore fitness function with default parameters; the top-scoring solution was retained for each ligand. The ligand binding site was defined to encompass a 12 Å radius about the C^α^ atom of E152, located at the center of the negatively charged surface on the “N” face of the M1 protein. This binding site definition covers major grooves on this side, as well as the hydrophobic pocket on the “H” face. Up to ten docking runs were performed for each ligand, with early termination enabled such that docking terminated when the best three solutions found were all within 1.5 Å RMSD.

For the LOPAC library, a molecular weight filter was used to remove compounds larger than 500 Da. The top 20 highest-scoring compounds were considered, from which a final subset of ten were selected based on structural diversity (Supplementary Fig. 5). For the Maybridge library, the top 100 highest-scoring compounds were considered, from which a subset of seven were selected based on structural diversity; this list was further reduced by applying a ‘reactivity’ filter that removed compounds possessing a reactive thiol group, resulting in a final selection of four compounds (Supplementary Fig. 6). Selected compounds from the LOPAC and Maybridge libraries were subsequently ordered from MolPort.

### Blind Docking of PHE

A blind docking technique was used to search for alternative PHE binding sites on the M1 surface. GOLD was used to dock PHE to overlapping binding sites over the entire surface of the N^1–164^ region of an M1 monomer (‘B’ chain of PDB entry 1EA3 with modified Q158 side chain as described above). A total of 41 potential binding sites were defined, each encompassing a 12 Å radius about the C^α^ atom of an amino acid residue whose side chain is a) basic, b) acidic, or c) hydrophobic and at the center of a surface-exposed hydrophobic patch or pocket (Supplementary Table 1). As such, these residues are important contributors to potential small-molecule binding “hot spots”. Ten docking runs were performed for PHE at each site and with four different scoring functions available in GOLD: ASP^52^, Chemscore^53^,^54^, Goldscore^51^,^55^ and ChemPLP^56^. The best-scoring docked PHE solution for each combination of site and scoring function was selected for further analysis. For each scoring function, the PHE-bound sites were ranked from best to worst, with 1 being the best. To remove the influence of bias in any given scoring function, a consensus rank was also assigned to each site by summing the ranks of the four individual scoring functions and ranking the resulting sums in ascending order (Supplementary Table 1). The top-ranked docked solutions and their corresponding M1 binding sites are depicted in Supplementary Figs 8 and 9.

### *In vitro* Inhibition of Virus Replication

Madin-Darby Canine Kidney (MDCK) epithelial cells (80–90% confluence) were infected with H1N1 A/WSN/33 (WSN/33) at indicated multiplicity of infection (MOI) for 1 h. After removal of inoculum, MDCK cells were incubated with Opti-MEM medium containing 1 μg/ml of TPCK-treated trypsin and test compounds at various concentrations at 33 °C, 5% CO_2_. Forty eight hours later, supernatants were harvested for HA titer determination using 0.5% turkey erythrocytes. The ability of test compounds to inhibit WSN/33 plaque formation was determined using an improved plaque assay^57^. Briefly, MDCK cells confluent in 12-well plates were inoculated with 100 PFU WSN/33 per well at 33 °C, 5% CO_2_ for 1 h. After the virus inoculum was removed, MDCK monolayers were overlaid with 1.2% Avicel containing 1 μg/ml of TPCK-treated trypsin with or without testing compounds and were incubated without disturbance for another 48 h to allow plaque formation. MDCK monolayers were then fixed with methanol and WSN/33 plaques were counted after staining with 1% crystal violet. In a separate experiment, cell viability after compound treatment was determined using the MTT assay.

### Surface Plasmon Resonance (SPR)

The interaction of M1 with M1 in the presence and absence of PHE was studied using a Reichert SR7500DC optical biosensor. NeutrAvidin sensor chips (Reichert Technologies, Depew, NY, USA) were used for capturing biotinylated M1 and Scrubber (Version 2.0c, 2008, BioLogic Software) was used for processing the data. Two biotinylated M1 protein chips were prepared utilizing recombinant M1 protein expressing the N^1-165^-domain of WSN/33^29^ and a biotin-avidin immobilization strategy. One chip was used to study M1–PHE interaction. Biotinylated M1 protein (50 μM) in 10 mM HEPES buffer, pH 7.4 containing 150 mM NaCl, 3.4 mM EDTA, and 0.05% Tween 80 was injected onto NeutrAvidin sensor chip for ~40 min with a constant flow of 5 μL/min at 10 °C. An immobilization level of ~700 μRIU was achieved. For M1–PHE interaction study, PHE at varying concentrations (1.67, 3.33, 12.5, 25 and 50 μM) was injected over the biotinylated M1 protein chip at flow rate of 20 μL/min and room temperature. Association and dissociation of PHE was monitored for 1–3 min. The increase in SPR signal was proportional to the PHE concentration. The association and dissociation rates were used to calculate the binding constant.

To study M1-M1 interaction, biotinylated M1 of 10 μM was injected over the NeutrAvidin sensor chip for ~5 min with a constant flow of 30 μL/min at 10 °C. An immobilization response of 150 μRIU was achieved. For the M1-M1 interaction study, M1 at 71, 143, 300 and 570 nM concentrations was injected as described above for M1–PHE interaction at flow rate of 30 μL/min and room temperature. Association and dissociation kinetics was monitored for over 5 min and the affinity was calculated by averaging the affinities at each concentration.

### Bio-layer Interferometry (BLI)

Octet Qke biolayer interferometer equipped with streptavidin biosensor tips (ForteBio, Inc., Menlo Park, CA, USA) was also used to assess the effects of PHE on the M1-M1 interactions. Biotinylated M1 was loaded onto streptavidin biosensor tips in pH 7.4 PBS solution containing 0.01% BSA and 0.002% Tween-20 (kinetics buffer) until the response reached 1 nm. The biotinylated M1 coated biosensor tips were then dipped in pH 7.4 kinetics buffer containing 10 μM unbiotinylated M1 with or without PHE at various concentrations for 1200 seconds. The entire measure cycle was maintained at 30 °C with orbital shaking at 1000 rpm.

### PHE Toxicity and Inhibition on *in ovo* Virus Replication

All 10-day-old embryonic eggs were specific-pathogen-free and were candled to ensure viability and good quality before each inoculation. A DMSO solution of PHE in pH 7.4 PBS (1:25 v/v, final DMSO level was 4%) was injected into 10-day-old embryonic eggs at 0.1 ml/egg. The doses of PHE tested included 150, 372, 554 and 740 ng/g, which was normalized based on the average weight of 10-day-old embryonic eggs. Eggs injected with 0.1 ml/egg of vehicle (DMSO diluted in pH 7.4 PBS, 1:25 v/v) served as controls. Each PHE dose was tested in 5 eggs. Injected eggs were then incubated at 37 °C for three days. The viability of embryos was monitored daily. In separate experiments, representative IAV strains H1N1 A/WSN/33 (WSN/33), pandemic H1N1 A/Maryland/13/2012 (pdm H1N1 MD/12), H3N2 A/Switzerland/9715293/2013 (SWZ/13) and A/Fiji/2/2015 (Fiji /15), and H5N1 vaccine reassortants A/Indonesia/05/2005XPR8 (IN/05XPR8) and A/Egypt/N03072/2010XPR8 (EG/10XPR8) (both reassortants have the multibasic cleavage motif removed from H5 HA and are in A/PR8/34 backbone) were co-injected with PHE at different concentrations into 10-day-old embryonic eggs. Three days later, allantoic fluids were harvested from individual eggs and were tested for HA titers using 0.5% turkey erythrocytes (H1N1, pdm H1N1 and H5N1) or 0.75% guinea pig erythrocytes (H3N2)^3^.

### Transmission Electron Microscopy (TEM)

WSN/33 was co-injected into 10-day-old embryonic eggs with or without PHE. Allantoic fluids were harvested and were subjected to purification by ultracentrifugation (30,000 rpm × 90 min, 4 °C)^24^. Purified viruses were fixed with 2% paraformaldehyde and 2% glutaraldehyde in PBS pH7.3 at room temperature overnight followed by three brief washes in PBS buffer and then post-fixation with 1% osmium tetroxide for another 1 h. After dehydration and infiltration, fixed viruses were embedded in epoxy resin and were subjected to ultra-microtome cutting. Ultrathin sections were then stained with uranyl acetate and lead citrate and were examined under a Zeiss Libra 120 Plus transmission electron microscope. TEM images were acquired using a Gatan US1000XP digital camera. Approximately 100 virions per treatment were randomly counted under TEM and % of fully spiked, partially spiked and completely stripped virions were determined. The M1 layer thickness of individual virions was determined by averaging the measures at 3, 6, 9 and 12 o’clock under TEM respectively^24^. Eleven to twelve representative virions per treatment were analyzed.

### Statistical Analysis

One-way analysis of variance (ANOVA) was conducted using GraphPad Prism Version 6.02. A *P* value of < 0.05 was considered significant.

## Additional Information

**How to cite this article**: Mosier, P. D. *et al*. Broad Spectrum Anti-Influenza Agents by Inhibiting Self-Association of Matrix Protein 1. *Sci. Rep.* **6**, 32340; doi: 10.1038/srep32340 (2016).

## Supplementary Material

Supplementary Information
