# Supplementary material for: Broad Spectrum Anti-Influenza Agents by Inhibiting Self-Association of Matrix Protein 1
Source: Sci Rep. 2016 Aug 30;6:32340. doi: 10.1038/srep32340 (PMC5004101; doi:10.1038/srep32340)

Supplementary Information

Broad Spectrum Anti-Influenza Agents by Inhibiting Self-Association of Matrix Protein 1

Philip D. Mosier[1], Meng-Jung Chiang[2], Zhengshi Lin[2], Yamei Gao[2], Bashayer Althufairi[1], Qibing Zhou[1,3], Faik Musayev[1], Martin K. Safo[1], Hang Xie[2]*, Umesh R. Desai[1]*

[1]*Department of Medicinal Chemistry and Institute for Structural Biology, Drug Discovery and Development, School of Pharmacy, Virginia Commonwealth University, Richmond, Virginia, United States of America*

[2]*Division of Viral Products, Office of Vaccines Research and Review, Center for Biologics Evaluation and Research, United States Food and Drug Administration, Bethesda, Maryland, United States of America*

[3]*Department of Nanomedicine & Biopharmaceuticals, National Engineering Research Center for Nanomedicine, Huazhong University of Science and Technology, Wuhan, Hubei, China*

Correspondence to: *Email: urdesai@vcu.edu (URD); Hang.Xie@fda.hhs.gov (HX)

TABLE OF CONTENTS

Content	Page
Suppl. Fig. 1. ProfileGrid analysis of 742 IAV M1 sequences.	S2
Suppl. Fig. 2. Plot of overall sequence conservation among 742 IAV M1 sequences.	S16
Suppl. Fig. 3. ProfileGrid analysis of 1282 IAV M2 sequences.	S18
Suppl. Fig. 4. Plot of overall sequence conservation among 1282 IAV M2 sequences.	S24
Suppl. Fig. 5. Structures of LOPAC library virtual screening hits.	S25
Suppl. Fig. 6. Structures of Maybridge library virtual screening hits.	S26
Suppl. Table 1. Blind docking results for PHE.	S27
Suppl. Fig. 7. Concept of M1 disruption at potentially multiple sites.	S28
Suppl. Fig. 8. Multiple alternative binding sites for PHE.	S29
Suppl. Fig. 9. Disruption of M1 oligomerization by PHE via steric incompatibility.	S30
Suppl. Fig. 10. In vitro and *in ovo* toxicity of PHE.	S31
Suppl. Fig. 11. *In ovo* HA titer reduction by PHE for additional IAV strains.	S32

Supplementary Figure 1. JProfileGrid 2.0 analysis of 742 unique IAV M1 sequences from the NCBI Influenza Virus Resource Database. TH = Threshold cutoff values for cell coloring. SEQ = number of sequences.

	Posn	1	2	3	4	5	6	7	8	9	10	
	Major	M	S	L	L	T	E	V	E	T	Y	
	P1	M	S	F	L	T	E	V	E	T	Y	
ala	A							1	2	1		A
cys	C		1									C
asp	D											D
glu	E						741		739			E
phe	F			2								F
gly	G		1						1			G
his	H			1								H
ile	I				1							I
lys	K						1					K
leu	L			738	740			1				L
met	M	742										M
asn	N		1			1						N
pro	P					4						P
gln	Q											Q
arg	R											R
ser	S		739		1							S
thr	T					737				741		T
val	V			1				740				V
trp	W											W
tyr	Y										742	Y
gap	-											-

	**Posn**	**11**	**12**	**13**	**14**	**15**	**16**	**17**	**18**	**19**	**20**	
	**Major**	**V**	**L**	**S**	**I**		**P**	**S**	**G**	**P**	**L**	
	**P1**	**V**	**L**	**S**	**I**	**I**	**P**	**S**	**G**	**P**	**L**	
**ala**	**A**			1								**A**
**cys**	**C**								1			**C**
**asp**	**D**								1			**D**
**glu**	**E**											**E**
**phe**	**F**	1		3								**F**
**gly**	**G**								739			**G**
**his**	**H**											**H**
**ile**	**I**				732	350					1	**I**
**lys**	**K**											**K**
**leu**	**L**		741			1					739	**L**
**met**	**M**											**M**
**asn**	**N**					1						**N**
**pro**	**P**			2			738			740	2	**P**
**gln**	**Q**											**Q**
**arg**	**R**											**R**
**ser**	**S**			735			4	742		2		**S**
**thr**	**T**			1	3	1						**T**
**val**	**V**	741	1		7	388			1			**V**
**trp**	**W**											**W**
**tyr**	**Y**					1						**Y**
**gap**	**-**											**-**
	**Posn**	**21**	**22**	**23**	**24**	**25**	**26**	**27**	**28**	**29**	**30**	
	**Major**	**K**	**A**	**E**	**I**	**A**	**Q**	**R**	**L**	**E**	**D**	
	**P1**	**K**	**A**	**E**	**I**	**A**	**Q**	**K**	**L**	**E**	**D**	
**ala**	**A**		741			742						**A**
**cys**	**C**											**C**
**asp**	**D**			1							634	**D**
**glu**	**E**			741						742		**E**
**phe**	**F**								1			**F**
**gly**	**G**										43	**G**
**his**	**H**						1		1			**H**
**ile**	**I**				742				2			**I**
**lys**	**K**	742						52				**K**
**leu**	**L**						2		738			**L**
**met**	**M**											**M**
**asn**	**N**										10	**N**
**pro**	**P**											**P**
**gln**	**Q**						739					**Q**
**arg**	**R**							690				**R**
**ser**	**S**										54	**S**
**thr**	**T**		1									**T**
**val**	**V**											**V**
**trp**	**W**											**W**
**tyr**	**Y**										1	**Y**
**gap**	**-**											**-**

	Posn	31	32	33	34	35	36	37	38	39	40	
	**Major**	**V**	**F**	**A**	**G**	**K**	**N**	**T**	**D**	**L**	**E**	
	**P1**	**V**	**F**	**A**	**G**	**K**	**N**	**T**	**D**	**L**	**E**	
**ala**	**A**	1		730				74				**A**
**cys**	**C**											**C**
**asp**	**D**						1		741		4	**D**
**glu**	**E**					1					736	**E**
**phe**	**F**		742									**F**
**gly**	**G**				742						2	**G**
**his**	**H**											**H**
**ile**	**I**	4						7				**I**
**lys**	**K**					738	4					**K**
**leu**	**L**									742		**L**
**met**	**M**	1										**M**
**asn**	**N**						724		1			**N**
**pro**	**P**							1				**P**
**gln**	**Q**											**Q**
**arg**	**R**					3						**R**
**ser**	**S**			5			13					**S**
**thr**	**T**							658				**T**
**val**	**V**	736		7				2				**V**
**trp**	**W**											**W**
**tyr**	**Y**											**Y**
**gap**	**-**											**-**

	Posn	41	42	43	44	45	46	47	48	49	50	
	**Major**	**A**	**L**	**M**	**E**	**W**	**L**	**K**	**T**	**R**	**P**	
	**P1**	**A**	**L**	**M**	**E**	**W**	**L**	**K**	**T**	**R**	**P**	
**ala**	**A**	730										**A**
**cys**	**C**											**C**
**asp**	**D**											**D**
**glu**	**E**				742							**E**
**phe**	**F**		1									**F**
**gly**	**G**	1										**G**
**his**	**H**											**H**
**ile**	**I**		2				87					**I**
**lys**	**K**							742		1		**K**
**leu**	**L**		738	2			653					**L**
**met**	**M**			739								**M**
**asn**	**N**											**N**
**pro**	**P**										742	**P**
**gln**	**Q**			1								**Q**
**arg**	**R**									740		**R**
**ser**	**S**	4										**S**
**thr**	**T**		1				1		742	1		**T**
**val**	**V**	7					1					**V**
**trp**	**W**					742						**W**
**tyr**	**Y**											**Y**
**gap**	**-**											**-**

	Posn	51	52	53	54	55	56	57	58	59	60	
	Major	I	L	S	P	L	T	K	G	I	L	
	P1	I	L	S	P	L	T	K	G	I	L	
ala	A						1					A
cys	C											C
asp	D											D
glu	E											E
phe	F											F
gly	G								742			G
his	H											H
ile	I	740					1			709		I
lys	K							741				K
leu	L		742			742					741	L
met	M									22		M
asn	N											N
pro	P	1			733							P
gln	Q											Q
arg	R							1				R
ser	S			742	9							S
thr	T	1					740					T
val	V									11	1	V
trp	W											W
tyr	Y											Y
gap	-											-

	Posn	61	62	63	64	65	66	67	68	69	70	
	Major	G	F	V	F	T	L	T	V	P	S	
	P1	G	F	V	F	T	L	T	V	P	S	
ala	A							1				A
cys	C		1									C
asp	D											D
glu	E											E
phe	F		736		740							F
gly	G	742										G
his	H									1		H
ile	I			4								I
lys	K											K
leu	L		3				742			2		L
met	M											M
asn	N										3	N
pro	P									738		P
gln	Q											Q
arg	R											R
ser	S									1	739	S
thr	T					742		741				T
val	V		2	738					742			V
trp	W											W
tyr	Y				2							Y
gap	-											-

	Posn	71	72	73	74	75	76	77	78	79	80	
	Major	E	R	G	L	Q	R	R	R	F	V	
	P1	E	R	G	L	Q	R	R	R	F	V	
ala	A											A
cys	C											C
asp	D											D
glu	E	741										E
phe	F									741		F
gly	G	1		741								G
his	H						1					H
ile	I										12	I
lys	K											K
leu	L				741	1	1					L
met	M											M
asn	N											N
pro	P						1		2			P
gln	Q		2		1	741						Q
arg	R		740	1			738	742	740			R
ser	S						1			1		S
thr	T											T
val	V										730	V
trp	W											W
tyr	Y											Y
gap	-											-
	Posn	81	82	83	84	85	86	87	88	89	90	
	Major	Q	N	A	L	N	G	N	G	D	P	
	P1	Q	N	A	L	N	G	N	G	D	P	
ala	A			739							1	A
cys	C											C
asp	D					2				741		D
glu	E											E
phe	F											F
gly	G						742		742	1		G
his	H											H
ile	I											I
lys	K											K
leu	L				742							L
met	M											M
asn	N		729			724		742				N
pro	P										741	P
gln	Q	742										Q
arg	R											R
ser	S		7	3		14						S
thr	T		6			2						T
val	V											V
trp	W											W
tyr	Y											Y
gap	-											-

	Posn	91	92	93	94	95	96	97	98	99	100	
	Major	N	N	M	D		A	V	K	L	Y	
	P1	N	N	M	D	R	A	V	K	L	Y	
ala	A				1		741					A
cys	C											C
asp	D	1			737							D
glu	E											E
phe	F										1	F
gly	G				3	1						G
his	H											H
ile	I			7				5		1		I
lys	K					290			726			K
leu	L									738		L
met	M			734						3		M
asn	N	729	742		1							N
pro	P											P
gln	Q											Q
arg	R					451			16			R
ser	S	11					1					S
thr	T	1		1								T
val	V							737				V
trp	W											W
tyr	Y										741	Y
gap	-											-

	Posn	101	102	103	104	105	106	107	108	109	110	
	Major		K	L	K	R	E	I	T	F	H	
	P1	K	K	L	K	R	E	I	T	F	H	
ala	A											A
cys	C											C
asp	D						1					D
glu	E						741					E
phe	F									740		F
gly	G					1						G
his	H										735	H
ile	I			4				621				I
lys	K	318	742		742	2						K
leu	L			738				1				L
met	M							111				M
asn	N										3	N
pro	P											P
gln	Q											Q
arg	R	424				739						R
ser	S									1		S
thr	T								742			T
val	V							9				V
trp	W											W
tyr	Y									1	4	Y
gap	-											-

	Posn	111	112	113	114	115	116	117	118	119	120	
	Major	G	A	K	E	V	A	L	S	Y	S	
	P1	G	A	K	E	V	A	L	S	Y	S	
ala	A		738			1	603				1	A
cys	C									1		C
asp	D											D
glu	E	1			741							E
phe	F							1				F
gly	G	741			1				2			G
his	H							1				H
ile	I					133		2				I
lys	K			738								K
leu	L							738				L
met	M					2						M
asn	N								1			N
pro	P										1	P
gln	Q											Q
arg	R			4								R
ser	S		3				139		739		736	S
thr	T		1								4	T
val	V					606						V
trp	W											W
tyr	Y									741		Y
gap	-											-

	Posn	121	122	123	124	125	126	127	128	129	130	
	Major		G	A	L	A	S	C	M	G	L	
	P1	T	G	A	L	A	S	C	M	G	L	
ala	A	229		740		731						A
cys	C						3	742				C
asp	D											D
glu	E											E
phe	F											F
gly	G		742				12			740		G
his	H											H
ile	I								2		3	I
lys	K											K
leu	L				742				1		738	L
met	M								739			M
asn	N											N
pro	P										1	P
gln	Q											Q
arg	R											R
ser	S	1		2		5	727			2		S
thr	T	512				6						T
val	V											V
trp	W											W
tyr	Y											Y
gap	-											-

	Posn	131	132	133	134	135	136	137	138	139	140	
	Major	I	Y	N	R	M	G	T	V	T	T	
	P1	I	Y	N	R	M	G	T	V	T	T	
ala	A							137		51	95	A
cys	C											C
asp	D							1				D
glu	E											E
phe	F											F
gly	G						742					G
his	H											H
ile	I	742							4			I
lys	K				5							K
leu	L											L
met	M					742			1			M
asn	N			740						10		N
pro	P											P
gln	Q											Q
arg	R				737							R
ser	S			2						1	4	S
thr	T							604		680	643	T
val	V								737			V
trp	W											W
tyr	Y		742									Y
gap	-											-

	Posn	141	142	143	144	145	146	147	148	149	150	
	Major	E		A	F	G	L	V	C	A	T	
	P1	E	V	A	F	G	L	V	C	A	T	
ala	A		112	738						742	2	A
cys	C								741			C
asp	D											D
glu	E	742										E
phe	F				618		1					F
gly	G		98			742						G
his	H											H
ile	I						1	17			3	I
lys	K											K
leu	L				124		740	3				L
met	M							2			1	M
asn	N											N
pro	P											P
gln	Q											Q
arg	R											R
ser	S		24	2					1			S
thr	T			1							736	T
val	V		508	1				720				V
trp	W											W
tyr	Y											Y
gap	-											-

	Posn	151	152	153	154	155	156	157	158	159	160	
	Major	C	E	Q	I	A	D	S	Q	H	R	
	P1	C	E	Q	I	A	D	S	Q	H	R	
ala	A					740		100				A
cys	C	741										C
asp	D						740					D
glu	E		742				2					E
phe	F											F
gly	G											G
his	H			1					14	742		H
ile	I				740							I
lys	K										13	K
leu	L											L
met	M											M
asn	N				1							N
pro	P											P
gln	Q			741					728			Q
arg	R										729	R
ser	S	1				1		642				S
thr	T				1							T
val	V					1						V
trp	W											W
tyr	Y											Y
gap	-											-

	Posn	161	162	163	164	165	166	167	168	169	170	
	Major	S	H	R	Q	M		T	T	T	N	
	P1	S	H	R	Q	M	A	T	I	T	N	
ala	A	1					289	134	6	1		A
cys	C											C
asp	D											D
glu	E											E
phe	F											F
gly	G											G
his	H		739									H
ile	I					3	6	5	57	1		I
lys	K			1								K
leu	L				1	3						L
met	M					736	4					M
asn	N		1							1	742	N
pro	P	1								1		P
gln	Q		1		741							Q
arg	R			741								R
ser	S	740						2				S
thr	T						1	601	679	738		T
val	V						442					V
trp	W											W
tyr	Y		1									Y
gap	-											-

	Posn	171	172	173	174	175	176	177	178	179	180	
	Major	P	L	I	R	H	E	N	R	M	V	
	P1	P	L	I	R	H	E	N	R	M	V	
ala	A											A
cys	C											C
asp	D											D
glu	E						742					E
phe	F											F
gly	G											G
his	H					742						H
ile	I			742							1	I
lys	K				82							K
leu	L		742									L
met	M									742		M
asn	N							742				N
pro	P	742										P
gln	Q											Q
arg	R				660				742			R
ser	S											S
thr	T											T
val	V										741	V
trp	W											W
tyr	Y											Y
gap	-											-

	Posn	181	182	183	184	185	186	187	188	189	190	
	Major	L	A	S	T	T	A	K	A	M	E	
	P1	L	A	S	T	T	A	K	A	M	E	
ala	A		740				741		741			A
cys	C											C
asp	D						1					D
glu	E										742	E
phe	F											F
gly	G											G
his	H											H
ile	I	69			2	1						I
lys	K							739				K
leu	L	665										L
met	M	8								742		M
asn	N											N
pro	P		1									P
gln	Q											Q
arg	R							3				R
ser	S			742								S
thr	T		1		740	741						T
val	V								1			V
trp	W											W
tyr	Y											Y
gap	-											-

	**Posn**	**191**	**192**	**193**	**194**	**195**	**196**	**197**	**198**	**199**	**200**	
	**Major**	**Q**	**M**	**A**	**G**	**S**	**S**	**E**	**Q**	**A**	**A**	
	**P1**	**Q**	**M**	**A**	**G**	**S**	**S**	**E**	**Q**	**A**	**A**	
**ala**	**A**			742		3				741	718	**A**
**cys**	**C**											**C**
**asp**	**D**							1				**D**
**glu**	**E**				1			741				**E**
**phe**	**F**											**F**
**gly**	**G**				741							**G**
**his**	**H**								7			**H**
**ile**	**I**		7				2					**I**
**lys**	**K**								1			**K**
**leu**	**L**		1			1			1			**L**
**met**	**M**		719									**M**
**asn**	**N**						4					**N**
**pro**	**P**											**P**
**gln**	**Q**	742							733			**Q**
**arg**	**R**											**R**
**ser**	**S**					735	736				16	**S**
**thr**	**T**					3					4	**T**
**val**	**V**		15							1	4	**V**
**trp**	**W**											**W**
**tyr**	**Y**											**Y**
**gap**	**-**											**-**

	**Posn**	**201**	**202**	**203**	**204**	**205**	**206**	**207**	**208**	**209**	**210**	
	**Major**	**E**	**A**	**M**	**E**	**V**	**A**	**S**	**Q**	**A**	**R**	
	**P1**	**E**	**A**	**M**	**E**	**I**	**A**	**N**	**Q**	**A**	**R**	
**ala**	**A**		742				741			631		**A**
**cys**	**C**							1				**C**
**asp**	**D**	1			1							**D**
**glu**	**E**	740			741							**E**
**phe**	**F**											**F**
**gly**	**G**	1						8				**G**
**his**	**H**											**H**
**ile**	**I**			11		131						**I**
**lys**	**K**								6			**K**
**leu**	**L**					1						**L**
**met**	**M**			730								**M**
**asn**	**N**							126				**N**
**pro**	**P**											**P**
**gln**	**Q**								728			**Q**
**arg**	**R**								8		742	**R**
**ser**	**S**							605				**S**
**thr**	**T**							2		111		**T**
**val**	**V**			1		610	1					**V**
**trp**	**W**											**W**
**tyr**	**Y**											**Y**
**gap**	**-**											**-**

	Posn	211	212	213	214	215	216	217	218	219	220	
	Major	Q	M	V	Q	A	M	R	T	I	G	
	P1	Q	M	V	Q	A	M	R	T	I	G	
ala	A					742			135			A
cys	C											C
asp	D											D
glu	E											E
phe	F											F
gly	G										742	G
his	H	2			110							H
ile	I			19					1	623		I
lys	K	4										K
leu	L	1										L
met	M		742				742					M
asn	N											N
pro	P											P
gln	Q	732			631							Q
arg	R	3			1			742				R
ser	S								2			S
thr	T								604			T
val	V			723						119		V
trp	W											W
tyr	Y											Y
gap	-											-

	Posn	221	222	223	224	225	226	227	228	229	230	
	Major	T	H	P	S	S	S		G	L	K	
	P1	T	H	P	N	S	S	A	G	L	R	
ala	A							451				A
cys	C											C
asp	D											D
glu	E											E
phe	F											F
gly	G								740			G
his	H		722	2								H
ile	I											I
lys	K										582	K
leu	L									742		L
met	M											M
asn	N		3		137			9				N
pro	P		1	740								P
gln	Q		15									Q
arg	R		1		3						160	R
ser	S				602	731	742	2	2			S
thr	T	742				11		280				T
val	V											V
trp	W											W
tyr	Y											Y
gap	-											-

	Posn	231	232	233	234	235	236	237	238	239	240	
	Major	D	D	L	L	E	N	L	Q	A	Y	
	P1	D	N	L	L	E	N	L	Q	A	Y	
ala	A		2			1				625		A
cys	C											C
asp	D	719	674			10				1		D
glu	E	2				722						E
phe	F			1								F
gly	G	1				4				1		G
his	H						1				1	H
ile	I				113					1		I
lys	K					3	4					K
leu	L			741	629			742				L
met	M											M
asn	N	20	66				737			2		N
pro	P											P
gln	Q								742			Q
arg	R											R
ser	S									1		S
thr	T									110		T
val	V					2				1		V
trp	W											W
tyr	Y										741	Y
gap	-											-

	Posn	241	242	243	244	245	246	247	248	249	250	
	Major	Q	K	R	M	G	V	Q	M	Q	R	
	P1	Q	K	R	M	G	V	Q	M	Q	R	
ala	A											A
cys	C											C
asp	D		1									D
glu	E											E
phe	F											F
gly	G					742						G
his	H									4		H
ile	I								64			I
lys	K		658									K
leu	L						10		90			L
met	M				742		2		586			M
asn	N		80									N
pro	P											P
gln	Q	741						742		736	1	Q
arg	R	1	2	740						2	741	R
ser	S											S
thr	T		1									T
val	V						730		2			V
trp	W			2								W
tyr	Y											Y
gap	-											-

	Posn	251	252			TH	SEQ
	Major	F	K			0	742
	P1	F	K			0.1	
						0.25	
ala	A			A		0.5	
cys	C			C		0.7	
asp	D			D		0.9	
glu	E		1	E			
phe	F	742		F			
gly	G			G			
his	H			H			
ile	I			I			
lys	K		737	K			
leu	L			L			
met	M			M			
asn	N			N			
pro	P			P			
gln	Q			Q			
arg	R		4	R			
ser	S			S			
thr	T			T			
val	V			V			
trp	W			W			
tyr	Y			Y			
gap	-			-			

Supplementary Figure 2. M1 sequence conservation across 742 unique IAV sequences from the NCBI Influenza Virus Resource Database. The M1 sequence is conserved near or at 100% at nearly all positions, except for a few (see Supplementary Figure 1).

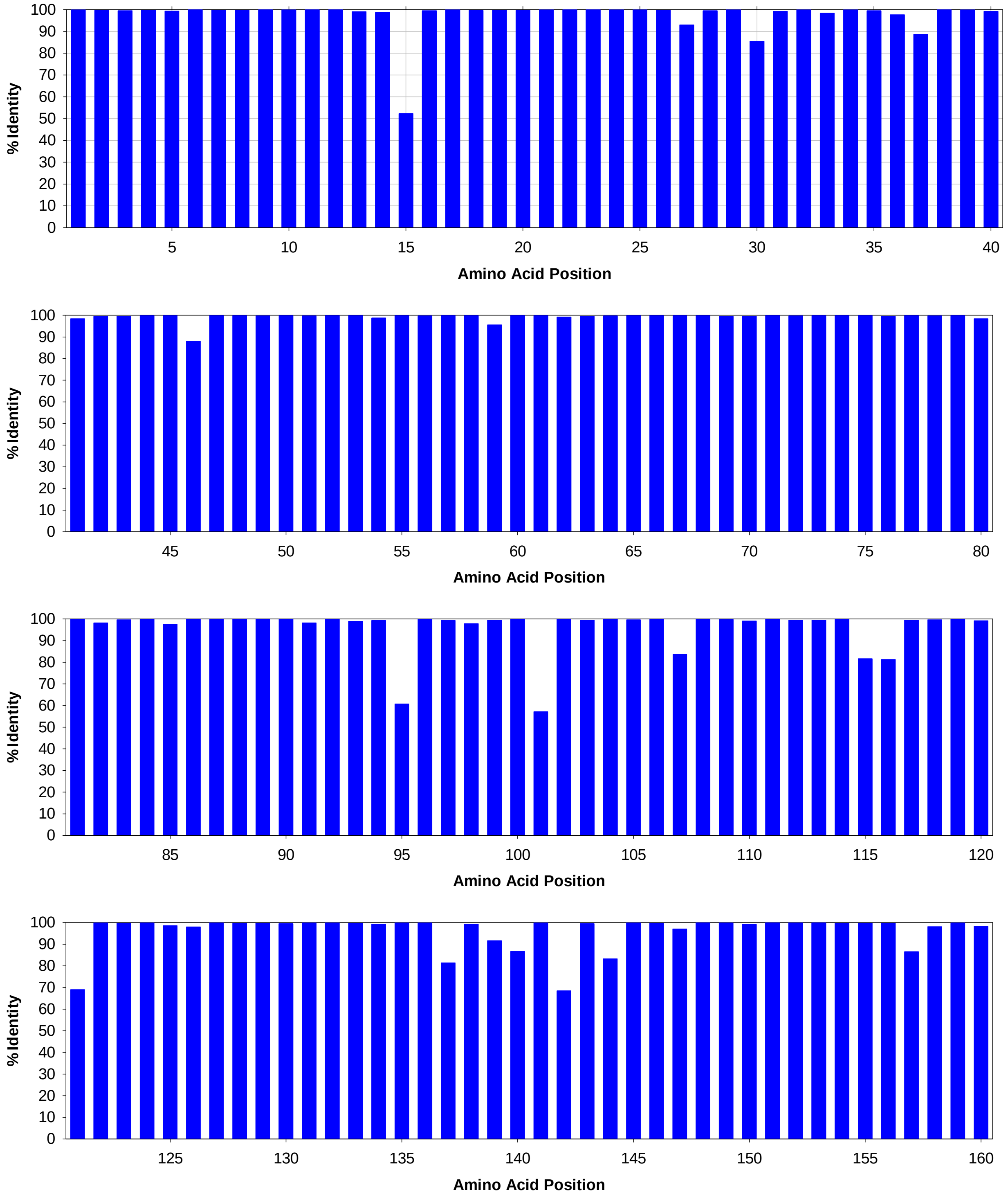

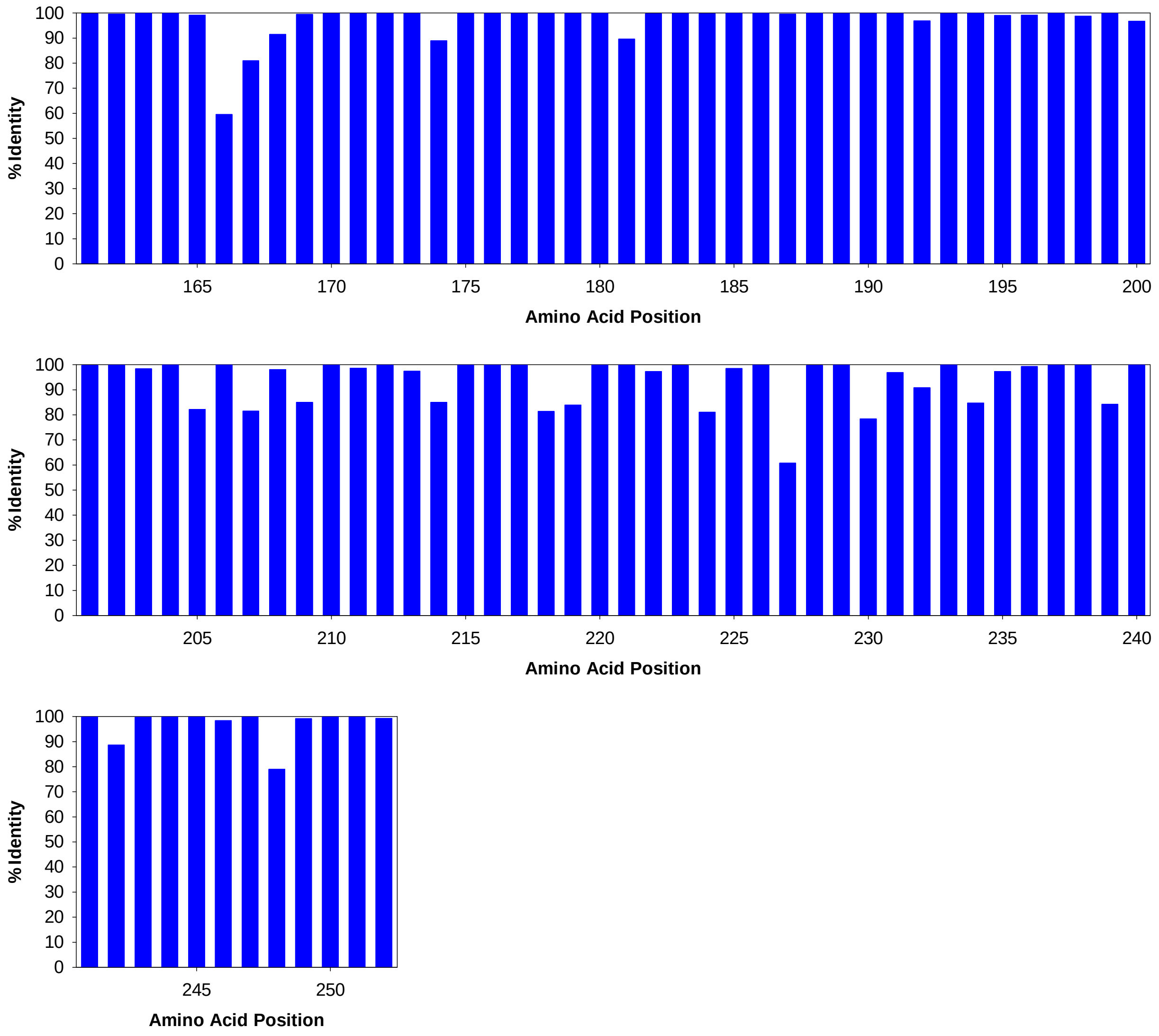

100
90
80
70
60
50
40
30
20
10
0
% Identity
165
170
175
180
185
190
195
200
Amino Acid Position
100
90
80
70
60
50
40
30
20
10
0
% Identity
205
210
215
220
225
230
235
240
Amino Acid Position
100
90
80
70
60
50
40
30
20
10
0
% Identity
245
250
Amino Acid Position

Supplementary Figure 3. JProfileGrid 2.0 analysis of 1282 unique IAV M2 sequences from the NCBI Influenza Virus Resource Database. TH = Threshold cutoff values for cell coloring. SEQ = number of sequences.

	Posn	1	2	3	4	5	6	7	8	9	10	
	Major	M	S	L	L	T	E	V	E	T	P	
	P1	M	G	L	L	T	E	V	E	T	P	
ala	A							1	2	1	2	A
cys	C		1									C
asp	D											D
glu	E						1281		1279			E
phe	F			1								F
gly	G		1						1			G
his	H										56	H
ile	I				1						1	I
lys	K						1					K
leu	L			1281	1280			1			151	L
met	M	1282										M
asn	N		1			1						N
pro	P					4					1066	P
gln	Q											Q
arg	R										3	R
ser	S		1279		1						2	S
thr	T					1277				1281	1	T
val	V							1280				V
trp	W											W
tyr	Y											Y
gap	-											-

	Posn	11	12	13	14	15	16	17	18	19	20	
	Major	T	R	N		W	E	C		C		
	P1	I	R	N	E	W	G	C	R	C	N	
ala	A						1					A
cys	C							1273		1157		C
asp	D			2			1					D
glu	E				469		976					E
phe	F							1		2		F
gly	G		1		811		262				3	G
his	H			1								H
ile	I	370		2							7	I
lys	K		101	22					502		11	K
leu	L											L
met	M											M
asn	N			912					190		406	N
pro	P											P
gln	Q											Q
arg	R		1180	4					588		1	R
ser	S			169					2	1	854	S
thr	T	912		170								T
val	V				2		42					V
trp	W					1282						W
tyr	Y							8		122		Y
gap	-											-

	Posn	21	22	23	24	25	26	27	28	29	30	
	Major	D	S	S	D	P	L	V		A	A	
	P1	D	S	S	D	P	L	V	V	A	A	
ala	A	3				27		107	45	1245	1272	A
cys	C											C
asp	D	1010		1	1238				106			D
glu	E				25				1			E
phe	F					2	5		30			F
gly	G	231		4				3	2			G
his	H					2						H
ile	I					4	71	161	485	1		I
lys	K											K
leu	L		12			9	1205	2	14			L
met	M							1	2			M
asn	N	1		60	18				9			N
pro	P					1191						P
gln	Q					1						Q
arg	R											R
ser	S		1270	1217		10		1		3	3	S
thr	T					9		48	62	17	5	T
val	V	37				27	1	959	522	16	2	V
trp	W											W
tyr	Y								4			Y
gap	-											-

	Posn	31	32	33	34	35	36	37	38	39	40	
	Major		I	I	G	I	L	H	L	I	L	
	P1	N	I	I	G	I	L	H	L	I	L	
ala	A											A
cys	C											C
asp	D	2										D
glu	E				1							E
phe	F								1		1	F
gly	G	1			1281							G
his	H							1281				H
ile	I		1226	1263		1275				1245		I
lys	K											K
leu	L			3			1259		1279		1281	L
met	M								1	10		M
asn	N	598										N
pro	P								1			P
gln	Q											Q
arg	R	1						1				R
ser	S	680										S
thr	T			2						18		T
val	V		56	14		7	23			9		V
trp	W											W
tyr	Y											Y
gap	-											-
	Posn	41	42	43	44	45	46	47	48	49	50	
	Major	W	I	L	D	R	L	F	F	K	C	
	P1	W	I	L	D	R	L	F	F	K	C	
ala	A			5								A
cys	C					1			1		1150	C
asp	D				1276							D
glu	E											E
phe	F			24			2	1268	1220		27	F
gly	G											G
his	H					7						H
ile	I		1266	34			2					I
lys	K									1281		K
leu	L			1147			1278	14	8			L
met	M		1									M
asn	N				3							N
pro	P											P
gln	Q									1		Q
arg	R	1				1273						R
ser	S		3	4		1			53		23	S
thr	T		7	68								T
val	V		5		2							V
trp	W	1281									1	W
tyr	Y				1						81	Y
gap	-											-

	**Posn**	**51**	**52**	**53**	**54**	**55**	**56**	**57**	**58**	**59**	**60**	
	**Major**	**I**	**Y**	**R**	**R**		**K**	**Y**	**G**	**L**	**K**	
	**P1**	**V**	**Y**	**R**	**L**	**F**	**K**	**H**	**G**	**L**	**K**	
**ala**	**A**	4										**A**
**cys**	**C**		12		12	1		3				**C**
**asp**	**D**							4	6			**D**
**glu**	**E**						13		12		1	**E**
**phe**	**F**		9		11	712		1		3		**F**
**gly**	**G**								1260			**G**
**his**	**H**		20	4	20			221			1	**H**
**ile**	**I**	1091			25	24				2		**I**
**lys**	**K**						1254				1156	**K**
**leu**	**L**	2			172	544		1		1274		**L**
**met**	**M**									1		**M**
**asn**	**N**		1					1			1	**N**
**pro**	**P**				1							**P**
**gln**	**Q**			4	2		1	2			116	**Q**
**arg**	**R**			1274	1026		13	1		1	7	**R**
**ser**	**S**		3		6				4	1		**S**
**thr**	**T**	3					1					**T**
**val**	**V**	182			6	1						**V**
**trp**	**W**											**W**
**tyr**	**Y**		1237		1			1048				**Y**
**gap**	**-**											**-**
	**Posn**	**61**	**62**	**63**	**64**	**65**	**66**	**67**	**68**	**69**	**70**	
	**Major**	**R**	**G**	**P**	**S**	**T**	**E**	**G**	**V**	**P**	**E**	
	**P1**	**R**	**G**	**P**	**S**	**T**	**E**	**G**	**V**	**P**	**E**	
**ala**	**A**				4	5	12		1		1	**A**
**cys**	**C**											**C**
**asp**	**D**										4	**D**
**glu**	**E**		1				1255				1256	**E**
**phe**	**F**				5							**F**
**gly**	**G**	27	1278				9	1281				**G**
**his**	**H**											**H**
**ile**	**I**	15							16			**I**
**lys**	**K**	19				2	6				19	**K**
**leu**	**L**			3					10			**L**
**met**	**M**	1				20			125			**M**
**asn**	**N**											**N**
**pro**	**P**			1278	1					1280		**P**
**gln**	**Q**											**Q**
**arg**	**R**	1214	2			1						**R**
**ser**	**S**	6	1		1271	1				2		**S**
**thr**	**T**			1		1251						**T**
**val**	**V**					2		1	1130		2	**V**
**trp**	**W**											**W**
**tyr**	**Y**				1							**Y**
**gap**	**-**											**-**

	Posn	71	72	73	74	75	76	77	78	79	80	
	Major	S	M	R	E	E	Y	R	Q	E	Q	
	P1	S	M	R	E	E	Y	R	K	E	Q	
ala	A	1										A
cys	C	1										C
asp	D				1							D
glu	E				1281	1277			23	1144		E
phe	F						3					F
gly	G			1		3						G
his	H								2			H
ile	I		1									I
lys	K					2			194	135	8	K
leu	L							1				L
met	M		1281									M
asn	N									2		N
pro	P											P
gln	Q							211	1061		1267	Q
arg	R			1281				1070	1	1	7	R
ser	S	1278										S
thr	T											T
val	V								1			V
trp	W											W
tyr	Y	2					1279					Y
gap	-											-

	Posn	81	82	83	84	85	86	87	88	89	90	
	Major	Q		A	V	D	V	D	D	G	H	
	P1	Q	N	A	V	D	A	D	D	S	H	
ala	A			1270	3		220		7			A
cys	C		1							4		C
asp	D		7	1		1257		1279	1214	27		D
glu	E				1	2			5			E
phe	F											F
gly	G		7		6	15		1	20	1024		G
his	H	2							1		1273	H
ile	I		6							1		I
lys	K	1										K
leu	L											L
met	M				1							M
asn	N		520			5			26	3	3	N
pro	P			1								P
gln	Q	1275									1	Q
arg	R	4								1	5	R
ser	S		741			3	1			220		S
thr	T			8				2	1			T
val	V			2	1271		1061		3	2		V
trp	W											W
tyr	Y								5			Y
gap	-											-

	Posn	91	92	93	94	95	96	97			TH	SEQ
	Major	F	V	N	I	E	L	E			0	1282
	P1	F	V	S	I	E	L	E			0.1	
											0.25	
ala	A		17			12			A		0.5	
cys	C								C		0.7	
asp	D			6		1			D		0.9	
glu	E					1108		1104	E			
phe	F	1280							F			
gly	G					5		7	G			
his	H								H			
ile	I		1		1267		1		I			
lys	K					8		170	K			
leu	L	2	1		1		1275		L			
met	M				4	4	3		M			
asn	N			1060					N			
pro	P						2		P			
gln	Q								Q			
arg	R				2	1			R			
ser	S		2	212	1		1		S			
thr	T			2	2			1	T			
val	V		1261		5	143			V			
trp	W								W			
tyr	Y			2					Y			
gap	-								-			

Supplementary Figure 4. M2 sequence conservation across 1282 unique IAV sequences from the NCBI Influenza Virus Resource Database. The overall degree of M2 conservation is less than in M1, and the fraction of M2 residues that are conserved at or near 100% is much less than in M1.

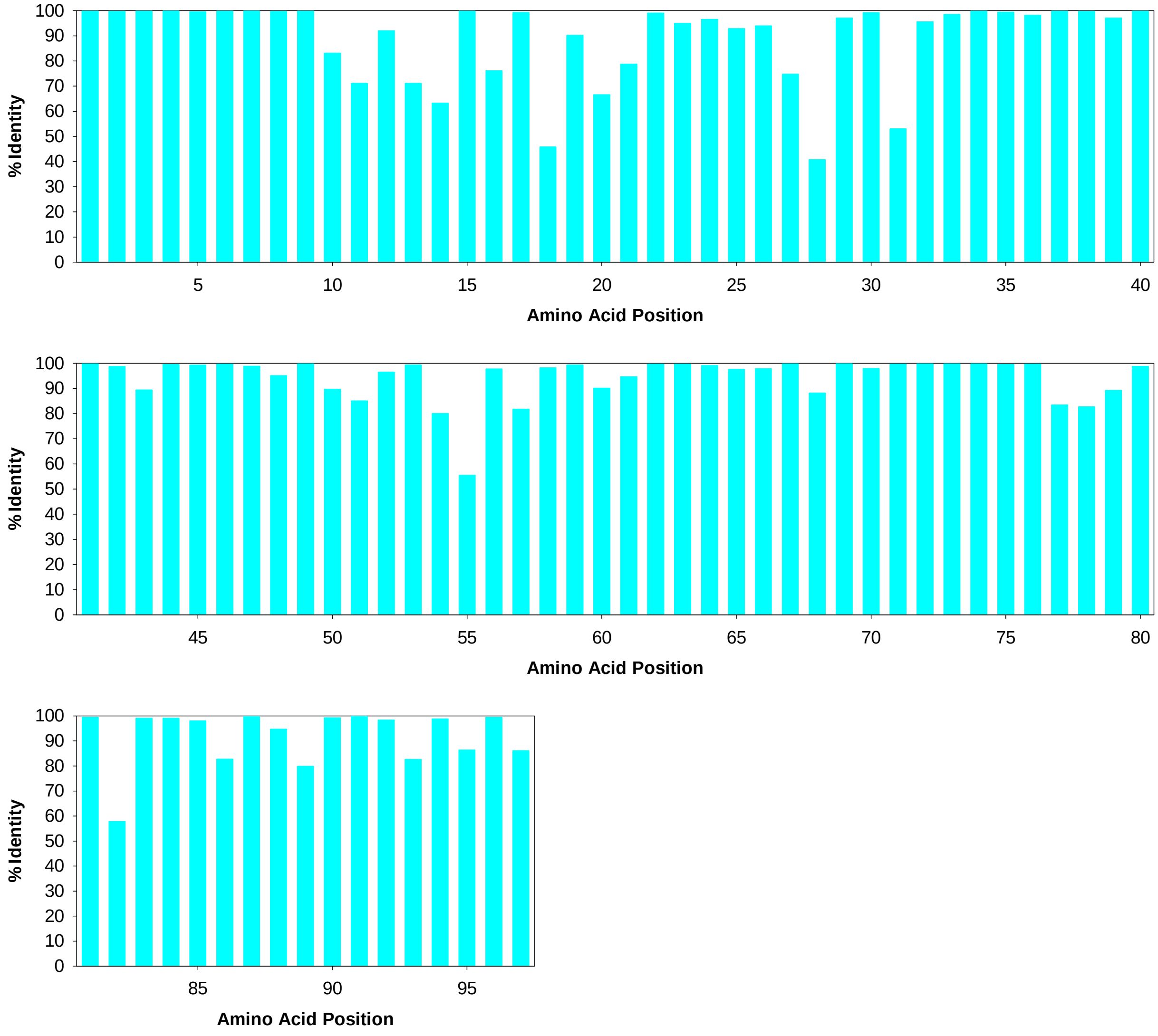

Supplementary Figure 5. The top ten 'hits' from the LOPAC library virtual screen.

	MET	MES	MIT	E64	PHE
**Structure**	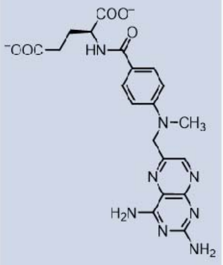	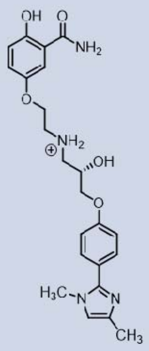	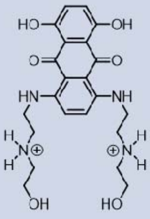	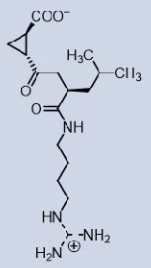	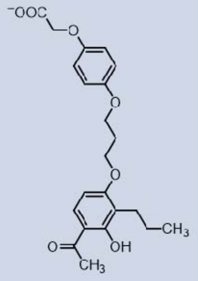
**Function**	Methotrexate: DHFR inhibitor	CGP-20712: β_1-Adrenergic antagonist	Mitoxantrone: Intercalating agent	E-64: Selective cysteine protease inhibitor	L-165041: PPAR-δ agonist
**ZINC ID**	ZINC01529323	ZINC03872010	ZINC03794794	ZINC04095645	ZINC02541693

	AMI	MRS	SAL	SKF	MIB
**Structure**	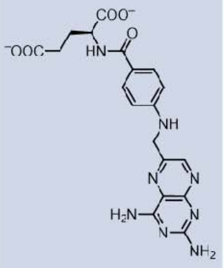	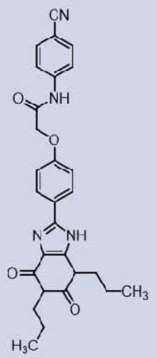	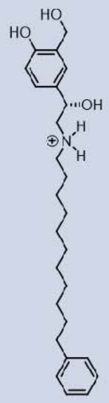	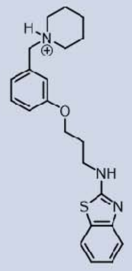	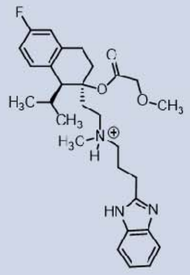
**Function**	Aminopterin: DHFR inhibitor	MRS-1754: Selective adenosine A2B antagonist	Salmeterol: Long-acting β-adrenergic agonist	Zolantidine: Histamine H2 antagonist	Mibefradil: Calcium channel blocker
**ZINC ID**	ZINC02036915	ZINC04475274	ZINC03785268	ZINC02386778	ZINC03782486

Supplementary Figure 6. The top four 'hits' from the Maybridge virtual screen.

	PDS	RDR	SPB	HTS
**Structure**	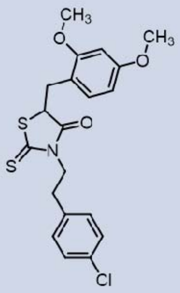	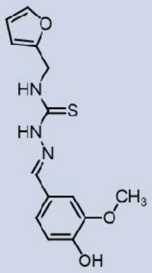	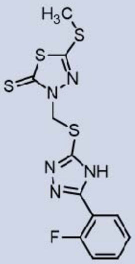	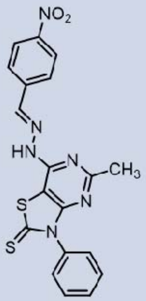
**ZINC ID**	ZINC13467571	ZINC12368009	ZINC04394522	ZINC08637248

Supplementary Table 1. Blind docking results for PHE.

Site	Central residue	ASP Rank	Chemscore Rank	Goldscore Rank	ChemPLP Rank	Total	Consensus Rank
1	K21	26	25 T	33	20	104	32
2	R27	6 T	14 T	8	5 T	33	7
3	K35	11 T	7 T	**3 T**	8	29	**4**
4	K47	29	15	20	2	66	18
5	R49	27	19	19	23	88	26
6	K57	—	—	—	—	—	—
7	R72	22	18	15	3 T	58	15
8	R76	15 T	5	11	**1 T**	32	**6**
9	R77	23	22	5 T	27	77	21
10	R78	25	21	5 T	22	73	20
11	K95	8	**1 T**	9	7	25	**2**
12	K98	9	**1 T**	14 T	6	30	**5**
13	R101	10	12	10	9 T	41	9 T
14	K102	2	17	14 T	26	59	16 T
15	K104	7	23	16	9 T	55	13
16	R105	20 T	33	29 T	34	116	33
17	K113	—	—	—	—	—	—
18	R134	12	2	7	**1 T**	22	**1**
19	L4	14	3	32	14 T	63	17 T
20	L12	17 T	27	21	24	89	27
21	I51	28	25 T	22	18	93	28
22	I107	31	35	29 T	33	128	34
23	Y119	4 T	11	23	25	63	17 T
24	L130	5	20	4	12	41	9 T
25	I154	21	9	28	21	79	22
26	E6	**1**	6	26	17	50	11
27	E8	3	13	34	32	82	24
28	E23	6 T	26	**1**	19	52	12
29	E29	16	29	3 T	11	59	16 T
30	D30	11 T	7 T	6	**4**	28	**3**
31	D38	11 T	24	3 T	5 T	43	10
32	E40	11 T	14 T	18	13	56	14
33	E44	19	30	24	10	83	25
34	E71	15 T	10	13	3 T	41	9 T
35	D89	30	34	2	15	81	23
36	D94	4 T	4	12	16	36	8
37	E106	20 T	32	17	29	98	29
38	E114	24	16	31	28	99	30 T
39	E141	18	8	30	14 T	70	19
40	E152	13	28	27	31	99	30 T
41	D156	17 T	31	25	30	103	31

Cell colors denote chemical nature of the central residue side chain: blue = basic; green = hydrophobic; red = acidic. Ties are denoted with the letter T. Numbers in red correspond to the docked poses of PHE at the top-ranked site for each scoring function combined with the top six consensus-ranked sites (highest-ranked scoring function selected). These eight poses identify four additional potential binding sites (due to binding site overlap) and are shown in **Supplementary Figures 8 and 9**).

Supplementary Figure 7. Cartoon representation of M1 disruption at potentially multiple sites.

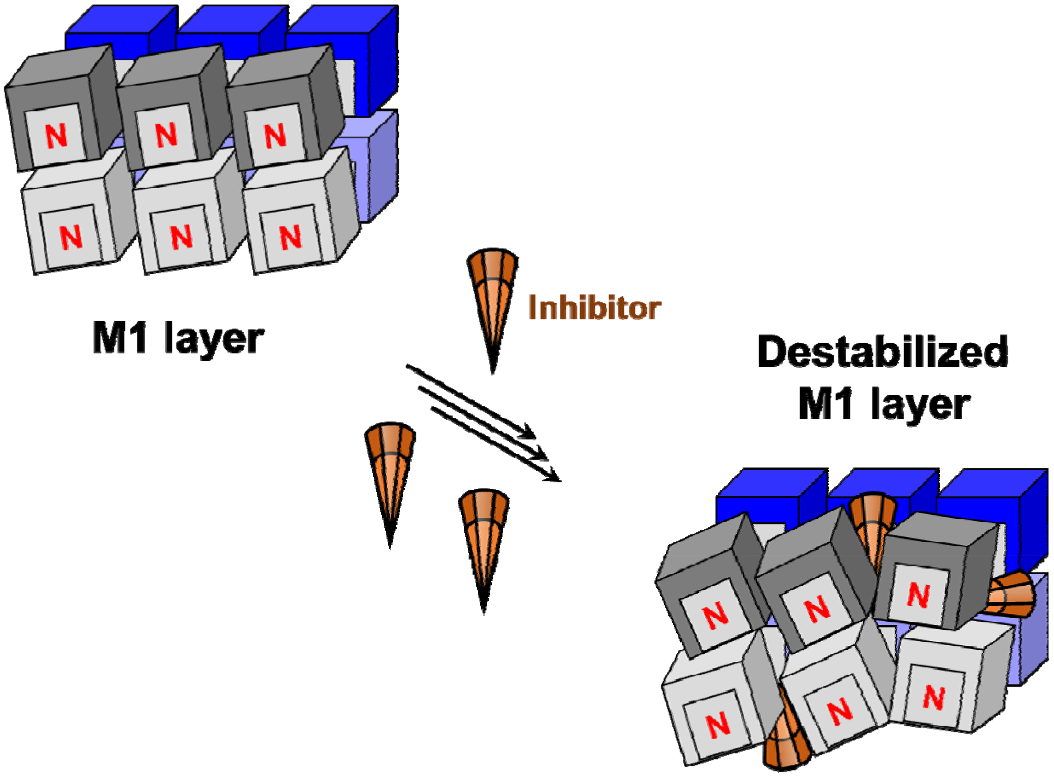

Supplementary Figure 8. Alternative binding sites for PHE. (a–b) Location of probable PHE binding sites on M1 corresponding to the top-ranked sites from blind docking studies (see **Supplementary Table 1**) with either (a) electrostatic or (b) hydropathic potential mapped onto the M1 surface. Color scheme is the same as Figure 1 in the main text. PHE with yellow carbons, initial top-ranked PHE pose from VS; PHE with white carbons, top-ranked blind docking poses.

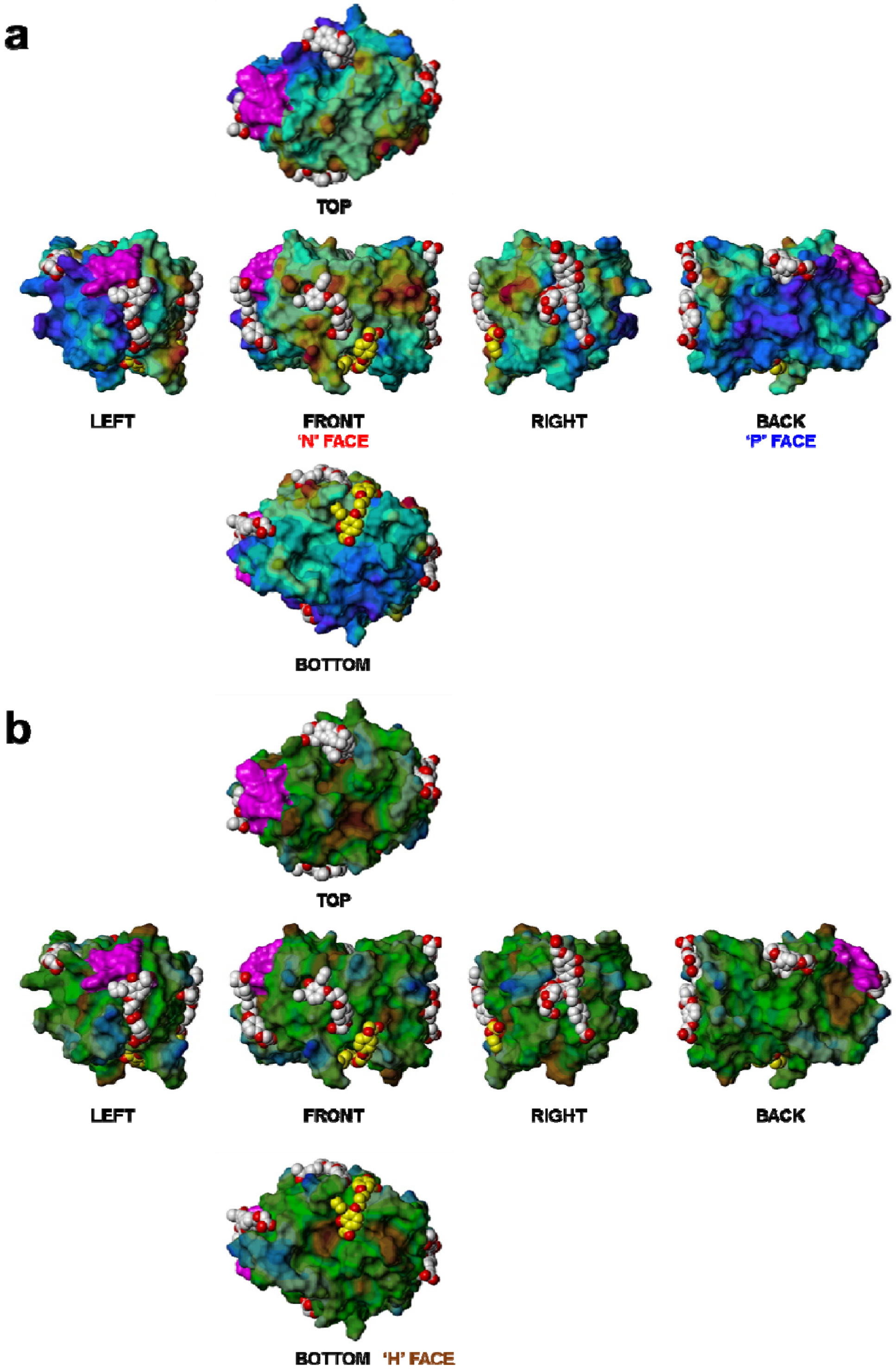

Supplementary Figure 9. The potential of PHE to disrupt M1 oligomerization at M1–M1 interfaces via steric incompatibility at multiple interaction sites, shown with top-ranked blind docking poses and within the context of the proposed oligomerization model described in Xie et al. 2013 and depicted in the lower-right cartoon. The docked poses introduce obvious steric clashes with adjacent subunits that would disrupt oligomerization. Ribbons are colored by subunit, as shown in the cartoon. Yellow Connolly surfaces represent PHE at the initial top-ranked pose from VS; other surfaces are colored by subunit.

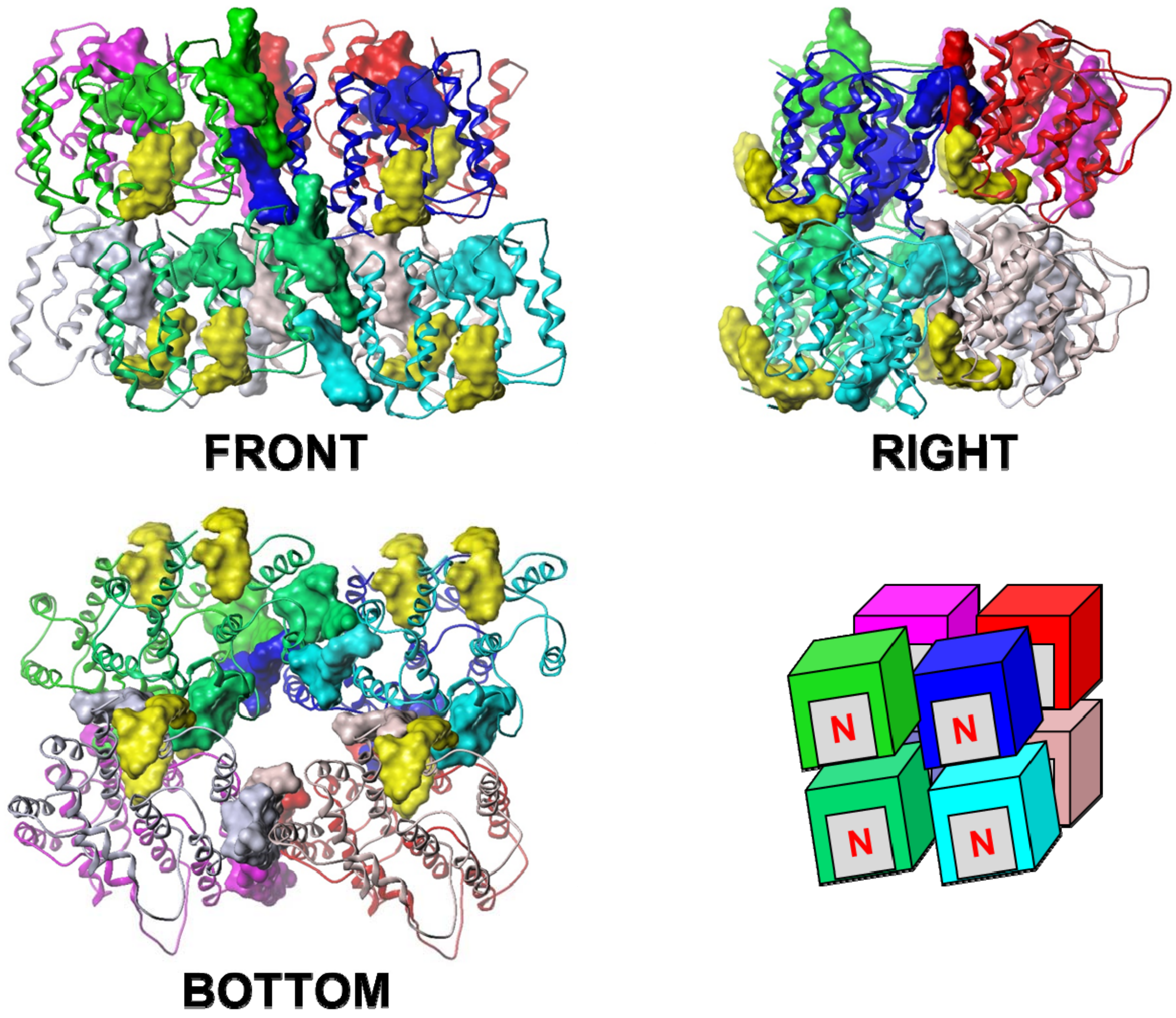

Supplementary Figure 10. In vitro and *in ovo* toxicity of PHE. A) PHE among six hit compounds had minimal cytotoxicity on MDCK cells (n=2 replicates). B) PHE at 740 ng/g had no toxicity on chicken embryos with or without the presence of viruses.

A

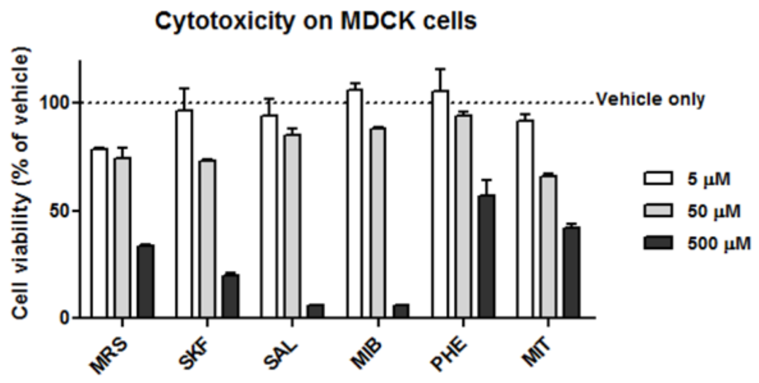

B

Embryo viability (live/total)					
PHE (740 ng/g) alone					
5/5					
PHE (740 ng/g) co-injected with the following viruses					
WSN/33 (H1N1)	MD/12 (pdm H1N1)	SWZ/13 (H3N2)	Fiji/15 (H3N2)	IN/05XPR8 (H5N1)	EG/10XPR8 (H5N1)
9/9	10/10	9/9	13/13	9/9	14/14

Supplementary Figure 11. PHE induction of a dose-dependent reduction in HA geometric mean of titer (GMT, black lines) of different IAV strains propagated in embryonic eggs, including A) H3N2 A/Fiji/2/2015 (Fiji/15) and B) H5N1 vaccine reassortant A/Egypt/N03072/2010XPR8 (EG/10XPR8). One-way ANOVA was performed to compare the differences between vehicle only and PHE treatments. HA titers were log-transformed before the analysis. * indicates P < 0.05; ** indicates P < 0.01; *** indicates P < 0.001.

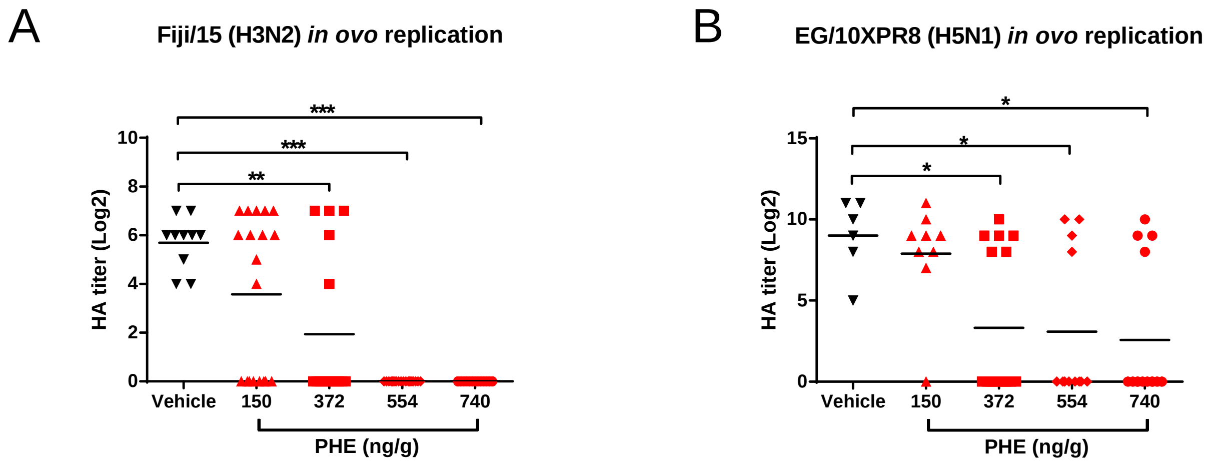

Supplement: Supplementary Information [file srep32340-s1.pdf]
